# Tandem Fusion of Hepatitis B Core Antigen Allows Assembly of Virus-Like Particles in Bacteria and Plants with Enhanced Capacity to Accommodate Foreign Proteins

**DOI:** 10.1371/journal.pone.0120751

**Published:** 2015-04-01

**Authors:** Hadrien Peyret, Annick Gehin, Eva C. Thuenemann, Donatienne Blond, Aadil El Turabi, Lucy Beales, Dean Clarke, Robert J. C. Gilbert, Elizabeth E. Fry, David I. Stuart, Kris Holmes, Nicola J. Stonehouse, Mike Whelan, William Rosenberg, George P. Lomonossoff, David J. Rowlands

**Affiliations:** 1 Department of Biological Chemistry, John Innes Centre, Norwich, United Kingdom; 2 School of Molecular and Cellular Biology, Faculty of Biological Sciences and Astbury Centre for Structural Molecular Biology, University of Leeds, Leeds, United Kingdom; 3 iQur Ltd, London, United Kingdom; 4 UK Division of Structural Biology, University of Oxford, Oxford, United Kingdom; Academia Sinica, TAIWAN

## Abstract

The core protein of the hepatitis B virus, HBcAg, assembles into highly immunogenic virus-like particles (HBc VLPs) when expressed in a variety of heterologous systems. Specifically, the major insertion region (MIR) on the HBcAg protein allows the insertion of foreign sequences, which are then exposed on the tips of surface spike structures on the outside of the assembled particle. Here, we present a novel strategy which aids the display of whole proteins on the surface of HBc particles. This strategy, named tandem core, is based on the production of the HBcAg dimer as a single polypeptide chain by tandem fusion of two HBcAg open reading frames. This allows the insertion of large heterologous sequences in only one of the two MIRs in each spike, without compromising VLP formation. We present the use of tandem core technology in both plant and bacterial expression systems. The results show that tandem core particles can be produced with unmodified MIRs, or with one MIR in each tandem dimer modified to contain the entire sequence of GFP or of a camelid nanobody. Both inserted proteins are correctly folded and the nanobody fused to the surface of the tandem core particle (which we name tandibody) retains the ability to bind to its cognate antigen. This technology paves the way for the display of natively folded proteins on the surface of HBc particles either through direct fusion or through non-covalent attachment via a nanobody.

## Introduction

**Fig 1:**
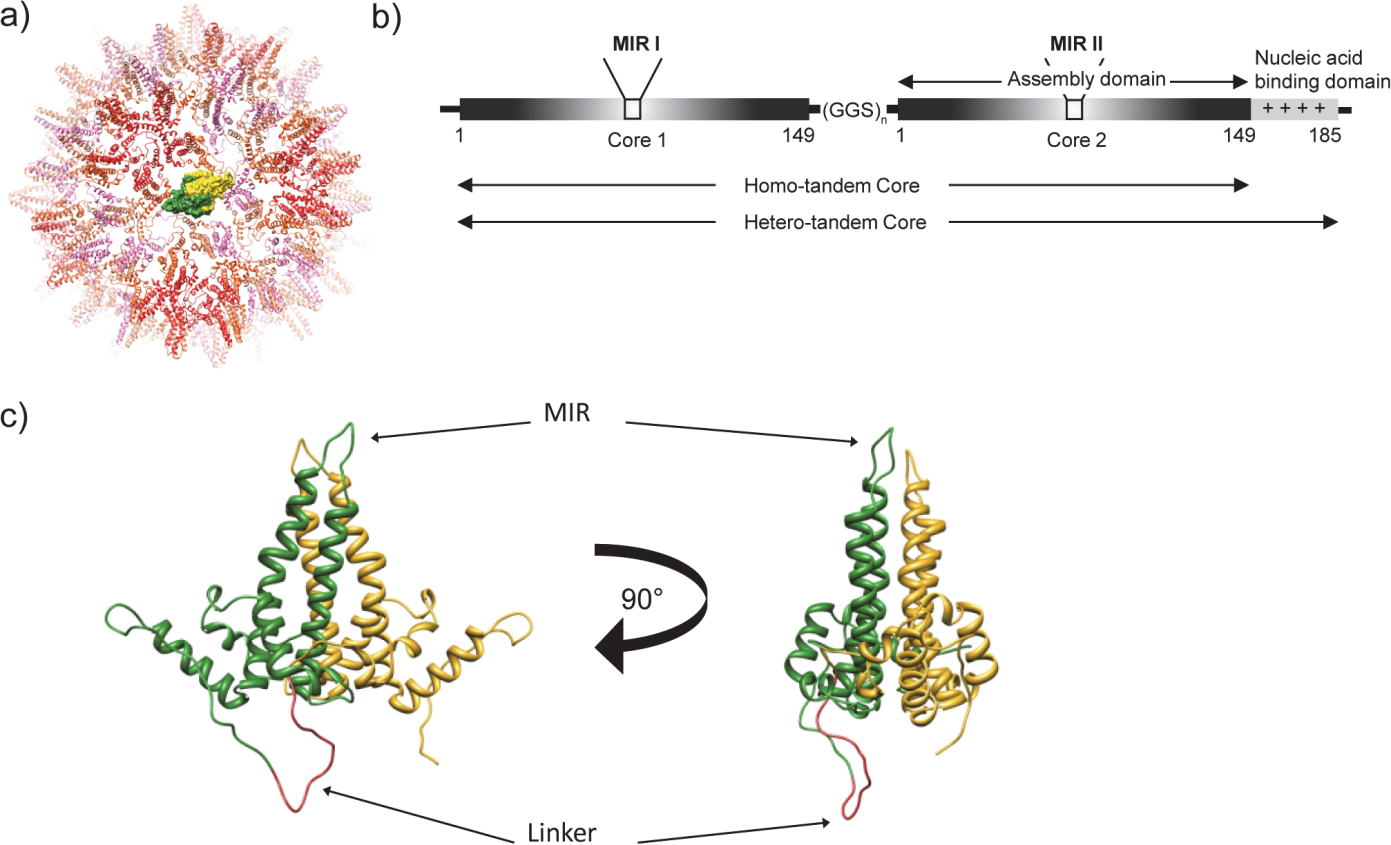
Tandem core technology. a) The structure of a monomeric HBc VLP with one HBcAg dimer shown in a surface representation coloured yellow and green. b) Two HBcAg sequences fused together via a flexible linker makes a tandem core construct, with either full-length (hetero-tandem) or truncated (homo-tandem) C-terminus, and two modifiable major insertion regions (MIRs). c) Structure of a tandem core protein: N-terminal core 1 (in green) is fused via a flexible linker (red) to C-terminal core 2 (yellow). The two views are related by a 90° rotation.

The core protein (HBcAg) of hepatitis B virus (HBV) readily assembles into highly immunogenic virus-like particles (VLPs) when expressed in either prokaryotic or eukaryotic expression systems [1–3]. These icosahedral hepatitis B core (HBc) particles are assembled from dimers of the 183–5 amino acid HBcAg protein and occur in two size classes with T = 3 or T = 4 symmetry, constructed from 90 and 120 dimeric subunits, respectively. The HBcAg protein has a high α-helical content and the dimer contacts form four-helix bundles which appear as prominent “spikes” at the surface of assembled particles. The structure of HBc particles has been determined by cryo-EM and X-ray crystallography [4–6] (Fig. 1A).

HBc particles have immunologically interesting properties: they induce very high titres of antibody, they can function as both T-cell independent and T-cell dependent antigens, and are potent activators of macrophages [7–9]. The HBcAg protein contains strong T cell epitopes and it has been proposed that the nucleic acid encapsidated within HBc VLPs further contributes to the immunogenicity of the particles via stimulation of Toll-like receptors [10,11]. Furthermore, third party antigens can be introduced at several positions in the HBcAg protein without compromising particle assembly. The most favourable of these sites is the major insertion region (MIR, or c/e1 loop) located at the tip of the α-helical spike [12,13]. Recombinant HBc particles bearing foreign antigenic sequences at this site induce strong immune responses to both HBc and the insert [14].

Early studies showed that HBc particles containing short foreign peptide sequences induced high serological responses to the inserted epitopes and, in the case of foot-and-mouth disease, solid protection of laboratory animals against virulent virus challenge was achieved [15]. More recently, HBc particles modified to display a malaria antigen were used in a phase I clinical trial [16,17]. Despite its apparent advantages, HBc has not been fully developed as a vaccine vector, in part because of limitations on the types of antigen that could be inserted. The preferred insertion site (the MIR), located at the tips of the surface-oriented spikes, inevitably presents two copies of the inserted sequence closely located in space, and so subject to potential steric clashes [12]. It has been demonstrated that the insertion of large or hydrophobic sequences into the MIR site of HBcAg protein can interfere with its ability to assemble into HBc particles with a consequent loss of antigenic and immunogenic properties [1].

A number of attempts have been made to overcome the structural barriers to recombinant chimaeric HBc production, including the co-expression of wild type HBcAg proteins and HBcAg with large inserts in the MIR site to create mosaics [18]. Another approach is the “SplitCore”, in which expression of the HBcAg protein as distinct N- and C- terminal portions allows assembly of structural dimers even in the absence of covalent linkage, thus allowing the inclusion of sequences tethered to the HBcAg protein by a single linkage [3]. In this manuscript, we present an additional solution to the problem with a construct termed tandem core [19]. In this system, two core proteins are joined together by a flexible linker to give a single fused dimer protein (Fig. 1B and C). We show that this dimer forms VLPs morphologically similar to those assembled from wild-type non-fused dimers when expressed in a variety of systems. This approach helps to reduce the limitations associated with insertion into the natural HBcAg protein since the assembly of the 4 helix bundle of the “spike” domain of the structural dimer is determined by covalent linkage and not random association of individual proteins. As a consequence, the tandem core system can allow for the insertion of a single large protein per “spike” or, potentially, for the dual expression of different sequences on each component of the “spike”. We show here that whole proteins may, indeed, be inserted into tandem cores without preventing their ability to assemble into VLPs. Furthermore we demonstrate that a single-domain antibody fragment (VHH, or nanobody) presented on the surface of tandem core particles through genetic fusion retains its ability to bind to its cognate antigen.

## Results

### Design of tandem cores

The crystal structure of the HBc particle (Fig. 1A) [6] was used to design a linker sequence predicted not to interfere with HBcAg protein dimer association and particle assembly. *In silico* analysis suggested that a 30 Å linker extending from amino acid 149 of the upstream copy to the N-terminus of the downstream copy should allow undistorted protein dimer association and result in assembly-competent covalently linked molecules. The GGS (glycine glycine serine) linkers were chosen as they are predicted to be flexible and structurally ‘neutral’ and so unlikely to distort protein folding and prevent particle formation. Initially we used 5 or 7 copies of the GGS sequence, each of which would fulfil the predicted minimum requirement of 30 Å length. The predicted model of two HBcAg proteins linked by a (GGS)_7_ is shown in Fig. 1C.

In addition to varying the number of GGS repeats linking the HBcAg monomers, we also produced alternative forms of the downstream molecule of the tandem pairs. This was because the C- terminal positively-charged domain is known to influence particle stability and assembly and to determine the content of nucleic acid incorporated into the assembled particles. In the hetero-tandem (He and the *E*. *coli* codon-optimised version CoHe) constructs the upstream copy is truncated at amino acid 149 whereas the downstream copy is full length. In the homo-tandem (Ho and the *E*. *coli* codon-optimised version CoHo) constructs both copies are truncated at amino acid 149.

### Expression of tandem core proteins in *E*. *coli*

**Fig 2:**
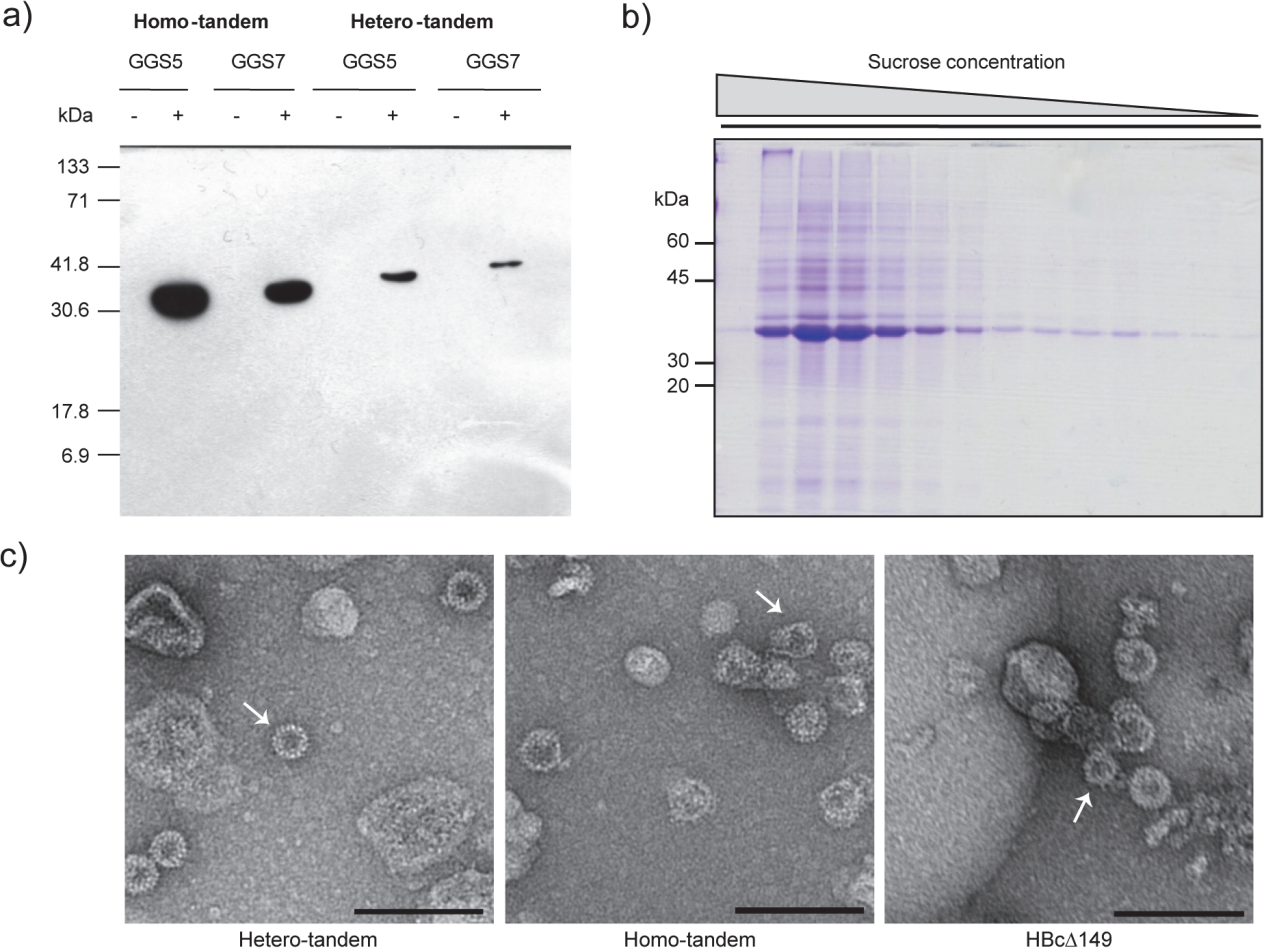
Tandem cores form VLPs when produced in *E*. *coli*. a) Western blot showing expression in induced (+) and uninduced (-) *E. coli* of homo- and hetero- tandem core with either 5 (GGS5) or 7 (GGS7) copies of the GGS sequence in the flexible linker between core 1 and core 2. b) Coomassie-stained gel of sucrose gradient fractions of CoHo (*E*. *coli* codon-optimised homo-tandem core with GGS7) produced in *E*. *coli*. The major band (fraction 2) reacted with anti-HBcAg antibody in western blot analysis. c) Electron micrographs of monomeric (HBcΔ149), codon-optimised homo-tandem (CoHo) and hetero-tandem (CoHe) core particles produced in *E*. *coli* and purified by sucrose gradient. Scale bar 100 nm. Arrows indicate smaller (T = 3) particles.

Constructs designed to express either the He or Ho proteins linked with 5 or 7 copies of GGS (He5, He7, Ho5, Ho7) were cloned into pTrc99A and expressed in *E*. *coli* JM109. Following induction with IPTG, bands corresponding to the anticipated sizes of He (38.8 and 41.0 kDa) and Ho (34.7 kDa and 36.6 kDa) tandem core proteins linked by either 5 or 7 GGS repeats, respectively, were detected on western blots of soluble bacterial extracts probed with the anti-HBcAg monoclonal antibody mAb 13, which recognises the MIR epitope. However, the level of protein expression from the Ho constructs was considerably higher than that obtained with the He constructs in the *E*. *coli* expression system (Fig. 2A), though both were <500 μg of recombinant protein per litre of bacterial culture. Similar differences in expression levels have been observed with the monomeric HBcAg protein upon removal of the C-terminal protamine-like region and are thought to be due to the inability of the truncated protein to bind RNA and so sequester its own mRNA [20]. Little difference was seen between tandem core constructs linked by 5 or 7 copies of GGS and most subsequent work was carried out using *E*. *coli* codon-optimised constructs (CoHe, CoHo) containing the GGS_7_ linker. The yield obtained with these constructs was approximately 1.25 mg/L for CoHe, and 0.6 mg/L for CoHo.

The particulate nature of the expressed tandem core proteins was investigated by sucrose density gradient centrifugation of clarified extracts of *E*. *coli* expressing the CoHo construct. Clarified bacterial extracts were concentrated by ammonium sulphate precipitation prior to analysis on continuous 15–45% (w/v) sucrose gradients. A broad peak of anti-HBcAg antibody—reactive material was detected in the bottom third of the gradient. SDS-PAGE analysis of the gradient fractions followed by Coomassie blue staining showed that the immunoreactive peak contained a major protein species corresponding in apparent molecular weight to the appropriate tandem core construct (Fig. 2B). Peak fractions from sucrose gradients were pooled, dialysed against 50 mM Tris/HCl pH 7.5, 100mM NaCl, negatively stained with uranyl acetate and examined by negative stain transmission electron microscopy. Isometric particles of 25–30 nm diameter and with morphology typical for HBcAg particles were seen (Fig. 2C). A majority of the particles appeared to be of the larger T = 4 type. However, smaller T = 3 particles were also visible (arrow). The surfaces of the particles were covered in small spikes, as is expected of HBc particles and it is interesting to note that the Ho cores are less well-structured than the He cores, as demonstrated by the more regular appearance of the He particles.

**Fig 3:**
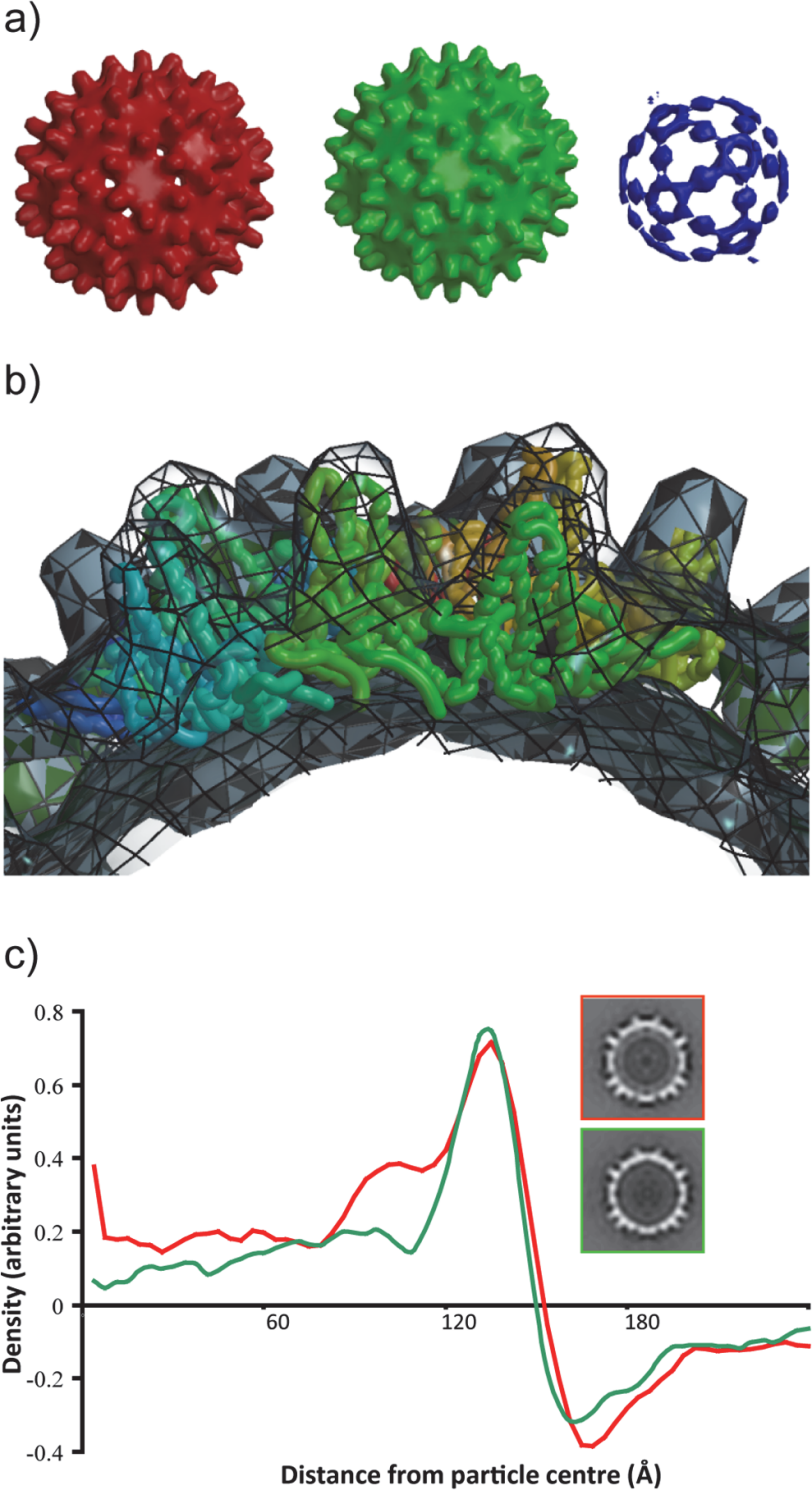
Cryo-electron microscopy analysis of E. coli- produced tandem core particles. a) Surface-rendered views of the reconstructions. Red—hetero-tandem core, contoured at 1σ. Green—homo-tandem core, contoured at 1σ. Blue—difference map, hetero-minus-homo, contoured at 4σ. b) Transverse view across 5-fold axis of the He core with co-ordinates from the HBc crystal structure (Wynne et al., 1999) fitted into the EM density. c) Density profiles of the He (red) and Ho (green) cores generated from translationally-aligned rotational averages. For comparison central sections of the He (upper panel) and Ho (lower panel) maps are shown to the right. A ring of density under the main capsid surface and at a radius of ~90 Å derives from the protamine-like region in He.

Cryo-electron microscopy and image analysis indicated that the populations of particles were predominantly T = 4, with a minority of smaller particles presumably possessing T = 3 symmetry. The smaller particles were excluded from the processing from the start; 99 images were incorporated into a final map of the Ho construct and 128 into a final map of the He construct. The core structures of both Ho and He VLPs were remarkably similar to the structure of the native core determined by cryo-EM and crystallography [6,21,22] with the spikes formed by a combination of two helices from each of the subunits in a dimer clearly visible on the core surfaces (Fig. 3A). Fitting of a portion of the atomic model of the core around a 5-fold icosahedral axis into the 3D reconstruction indicates the excellence of the agreement (Fig. 3B). The difference in density between the He and Ho cores derives from the presence of the protamine-like domain that binds nucleic acid. The additional density was also present in circularly averaged sums of the images used for each reconstruction (Fig. 3C).

### Expression of tandem core proteins in plants

**Fig 4:**
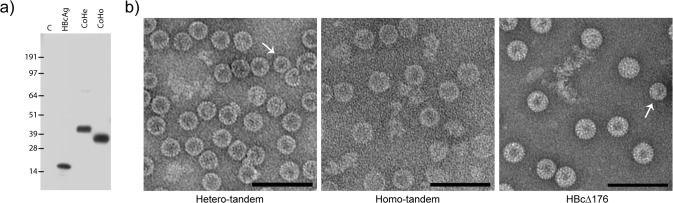
Tandem cores form VLPs when expressed in *N*. *benthamiana*. a) Western blot showing expression in *N*. *benthamiana* of monomeric (HBcAg), hetero-tandem (CoHe) and homo-tandem (CoHo) constructs. Lane C—empty vector control. b) Electron micrographs of monomeric (HBcΔ176), homo-tandem (CoHo) and hetero-tandem (CoHe) core particles produced in *N*. *benthamiana* and purified by sucrose gradient. Scale bar 100 nm. Arrows indicate smaller (T = 3) particles.

Tandem core constructs were expressed transiently in *Nicotiana benthamiana* plants using the pEAQ-*HT* expression vector [23,24]. Western blot analysis of total extracts of *N*. *benthamiana* leaves infiltrated with either a monomeric form of HBcAg (pEAQ-Δ176), a hetero-tandem (pEAQ-CoHe) or a homo-tandem construct (pEAQ-CoHo) using anti-HBcAg monoclonal antibody 10E11 (which recognises the N-terminus of HBcAg) revealed bands of the expected size (Fig. 4A), indicating that all the constructs expressed well in plants. To confirm that the expressed proteins could assemble into particles, clarified extracts were centrifuged on sucrose gradients and the distribution of HBcAg among the fractions examined by western blotting as above. It was noted that the peak from the plant-expressed CoHo construct was broad, in contrast to that from the CoHe construct which was present exclusively in the 30–40% sucrose fractions. Thus, as in *E*. *coli*, the CoHe construct produced more homogeneous particles than CoHo. It was estimated that the yield of particles from monomeric Δ176 approached 1 mg/g of fresh-weight tissue, and CoHe produced about 0.2 mg/g. To confirm particle formation, negatively-stained samples from the peak fractions were examined by transmission electron microscopy. This showed that both CoHe and CoHo proteins produced in plants assembled into particles of regular shape and size which resembled monomeric Δ176 HBc particles (Fig. 4B). In all three cases, a majority of the particles appeared to be of the larger T = 4 type, but smaller T = 3 particles were also visible (arrows). The plant-expressed particles resembled those expressed in *E*. *coli* from CoHo and CoHe constructs but appeared to be more homogeneous.

### Analysis of plant-derived hetero-tandem cores displaying GFP

**Fig 5:**
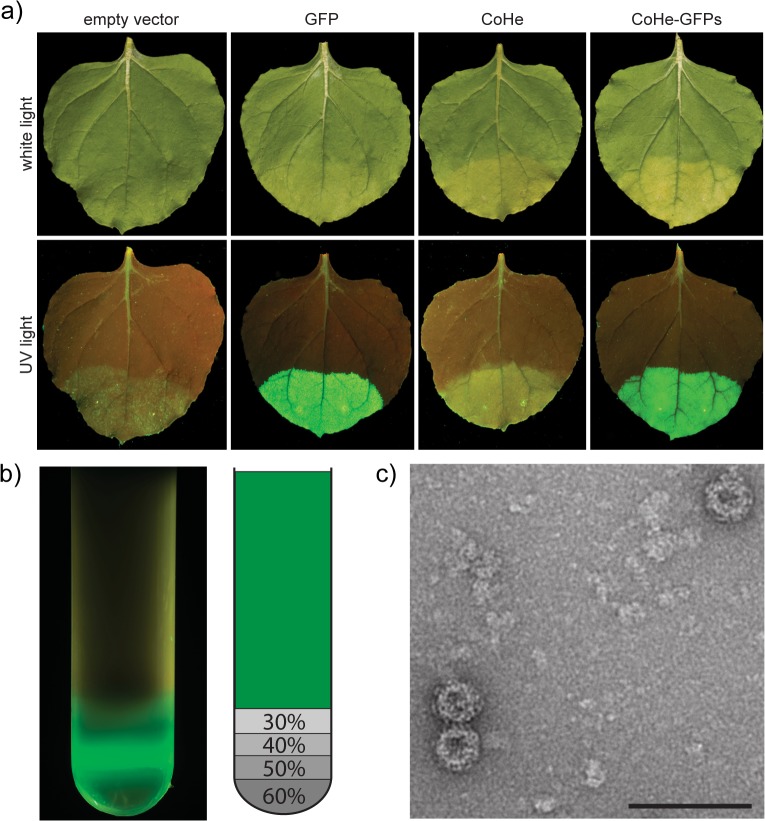
Tandem cores can display correctly-folded GFP in plants. a) White light (top) and UV light (bottom) images of *N*. *benthamiana* leaves expressing different constructs via the pEAQ-*HT* vector. b) UV light image of an ultracentrifuge tube after sucrose gradient purification of plant-produced CoHe-GFPs. The diagram on the right indicates the location of the sucrose layers and their concentration. The area represented in green is the clarified plant lysate. c) Electron micrograph of plant-produced CoHe-GFPs VLPs purified by sucrose gradient. Scale bar 100 nm.

To assess the ability of plants to express tandem cores with the sequence of GFP inserted in the MIR of core 2, *N*. *bethamiana* leaves were infiltrated with pEAQ-CoHe-GFPs (GenBank accession number KM396758). In this construct, the GFP is linked to core 2 with small (3 amino acid) linkers on either side. As controls, leaves were separately infiltrated with pEAQ-CoHe (no GFP), pEAQ-*HT*-GFP (expressing soluble GFP), or the empty pEAQ-*HT* vector control. Inspection of the leaves under ultraviolet illumination, revealed intense GFP fluorescence in those areas infiltrated with pEAQ-CoHe-GFPs or pEAQ-*HT*-GFP, but not with the constructs lacking the sequence of GFP (Fig. 5A). The fact that GFP inserted into CoHe retains its fluorescence indicates that it has folded correctly.

To further investigate the ability of GFP tandem cores to form particles, leaf tissue expressing CoHe-GFPs was harvested 7 days post-infiltration (dpi) and analysed on a 30–60% discontinuous sucrose gradient. Following ultracentrifugation, the tube was illuminated with UV light, revealing an intensely fluorescent band at the 40% sucrose boundary (Fig. 5B). No fluorescence was detected in the upper region of the gradient, indicating that the majority of the fluorescent material had assembled into particulate structures. Transmission electron microscopy confirmed the presence of regular HBc-like particles (Fig. 5C). Similar results were obtained with CoHe-GFPL, a construct in which the GFP insert is flanked by longer (10 aa) glycine-serine-rich linkers (see below). Overall a yield of approximately 10 μg purified particles per gram fresh weight of infiltrated tissue was obtained for each GFP-expressing tandem construct.

**Fig 6:**
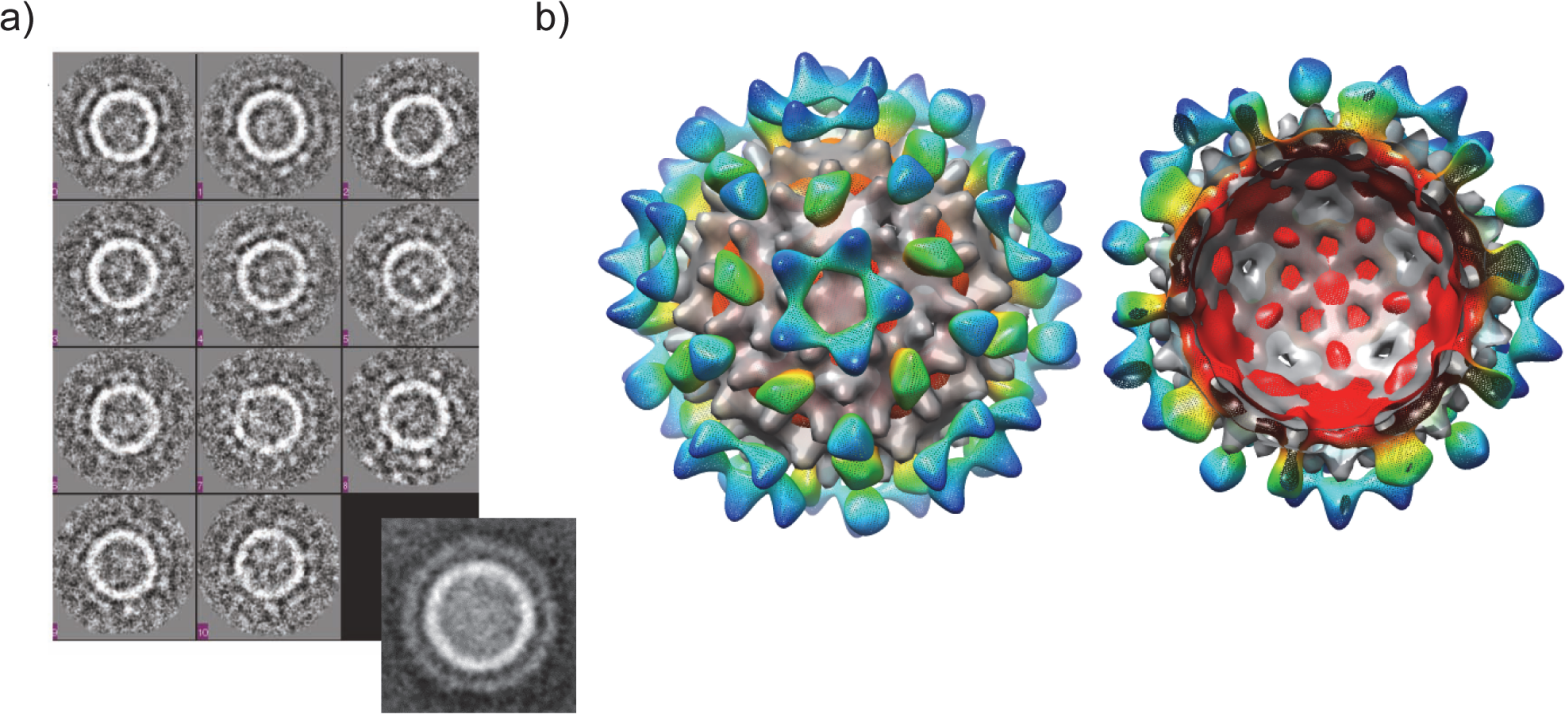
Cryo-EM analysis of plant-produced CoHe-GFPL VLPs. a) Particles were flash-frozen in vitreous ice, then subjected to cryo-electron microscopy. Class averages were obtained from 441 individual particles using EMAN software. The expanded view (lower right) is of an average of all images used. b) 3D reconstruction of the particles using icosahedral symmetry, superimposed on the He map as shown in Fig. 3. The CoHe-GFPL map is coloured red-to-blue from the centre of the volume towards its edge; the He map is shown in grey.

To gain insights into how GFP is presented on the surface of tandem cores, particles of CoHe-GFPL (at approx. 40 μg/ ml) were analysed by cryo-electron microscopy. 441 independent particles were picked from the CEM image and class averages produced with EMAN software (Fig. 6A). These were used to obtain a 3D reconstruction (Fig. 6B) which displays density projecting from the capsid surface which arises from the GFP. These data indicate that the GFP-bearing tandem core is able to assemble into particles with a core structure resembling that of wild-type HBcAg. The further layer of density surrounding the tandem core does not contain discrete regions deriving from each GFP attached to one MIR of every tandem core spike. Instead, and presumably due to flexibility in the linkers between the MIR and the GFP, an icosahedrally-averaged pattern of density is seen with the greatest concentration being over the 2-fold axes where apparent spikes arise from the capsid surface, and at around the 3-fold and 5-fold axes. Nevertheless, the presence of this density layer proves that the insertion in the MIR is presented on the outside of the particle, while it is clear that the presence of GFP does not prevent the formation of an HBV core.

### Use of tandem technology to display functional nanobodies

**Fig 7:**
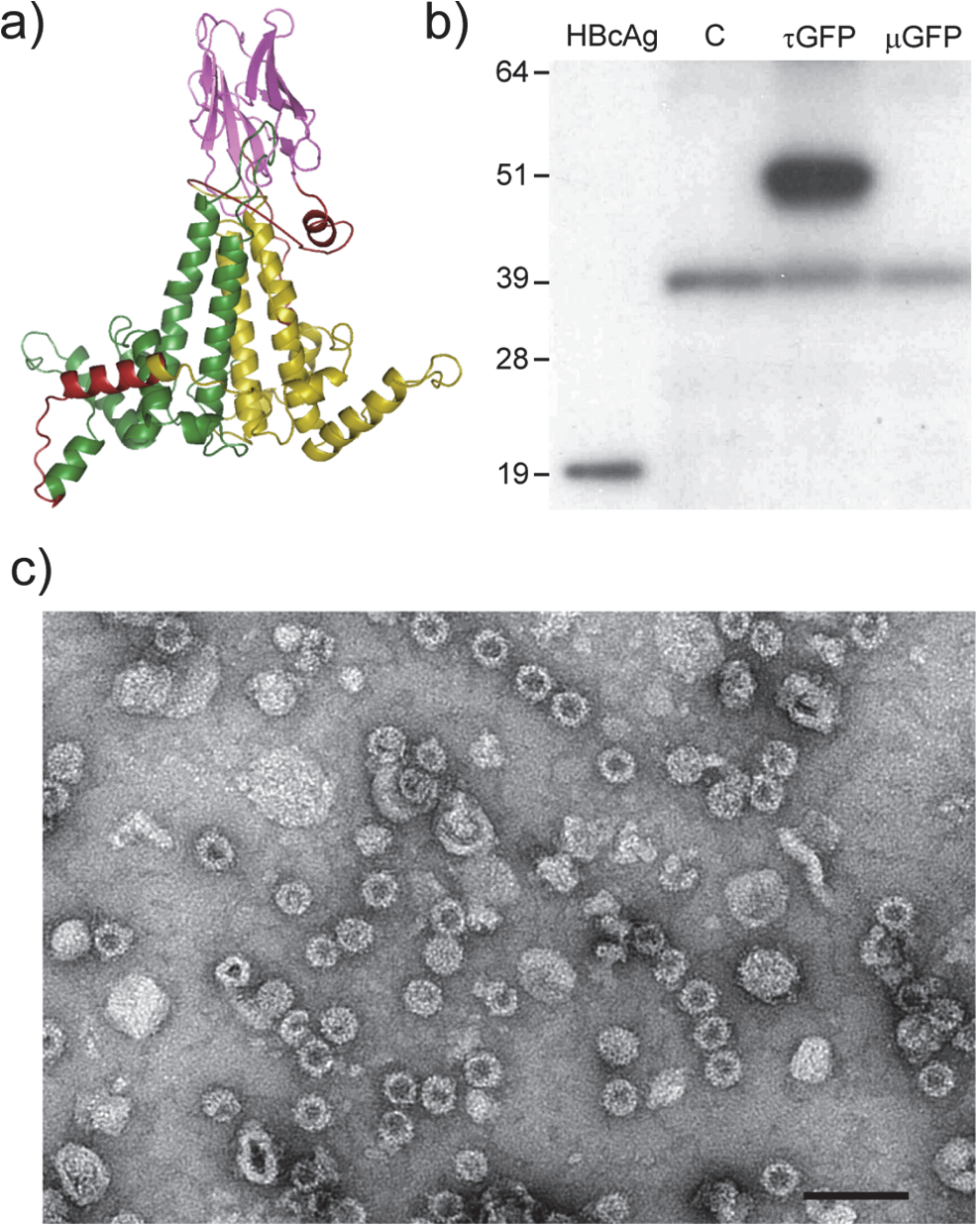
τGFP expressed in plants forms VLPs. a) Predicted structure of the τGFP tandibody protein (Swiss-Prot model): green: core 1, yellow: core 2, pink: anti-GFP nanobody, red: linkers. b) Western blot of crude plant extracts. C: empty vector control, τGFP: tandem HBcAg construct with anti-GFP VHH in the core 2 MIR, μGFP: monomeric HBcAg containing anti-GFP VHH in the MIR. The 39 kDa band found in all plant extracts is non-specific. c) Electron micrograph of plant-produced τGFP particles purified by sucrose cushion. Scale bar 100 nm.

The tandem HBcAg protein display technology was adapted to present a camelid single-domain antibody fragments (VHH, or nanobody) on the particle surface, yielding a novel type of HBc chimera which was termed a tandibody particle. The predicted structure of this construct is shown in Fig. 7A. To enable the facile insertion of the desired sequence, a new construct based on CoHe, named tEL, was constructed which had unique restriction sites in each MIR. This construct was inserted into the pEAQ-*HT* vector to give pEAQ-tEL. The 12 kDa anti-GFP “Enhancer” VHH [25] was inserted into the MIR of core 2 of pEAQ-tEL and the resulting construct designated pEAQ-τGFP (GenBank accession number KM396759) to refer to the two components: tandem HBcAg with a fused nanobody (τ, which stands for tandibody) with specificity to GFP, by analogy to the common abbreviation for an IgG specific to GFP: α-GFP. As a control, the same VHH sequence was also inserted into the MIR loop of a monomeric construct based on core 2 of pEAQ-t-EL, pEAQ-mEL, to give pEAQ-μGFP. The two constructs, together with a pEAQ-*HT* empty vector control were separately infiltrated into *N*. *benthamiana* leaves. Clarified protein samples from the infiltrated areas were prepared 7 dpi and analysed by western blotting using anti-HBcAg monoclonal antibody 10E11. The results showed the accumulation of HBcAg-specific material of the expected size (55 kDa) in leaves infiltrated with pEAQ-τGFP, while no protein corresponding to the expected size (37 kDa) could be detected in extracts from leaves infiltrated with pEAQ-μGFP (Fig. 7B). All samples, including those from leaves infiltrated with the empty vector, pEAQ-*HT*, showed a cross-reactive band of unknown origin at approximately 39 kDa. The failure to detect any material corresponding to μGFP is most likely due to the inability of this material to assemble into stable particles, resulting in its rapid turnover. Whatever the cause, these results demonstrated that a tandem construct, as opposed to a monomeric one, is required for the efficient display of VHH sequences. TEM analysis showed the presence of HBc-like particles (Fig. 7C) and the recovered yield of τGFP was estimated to be similar to that of CoHe-GFPs.

**Fig 8:**
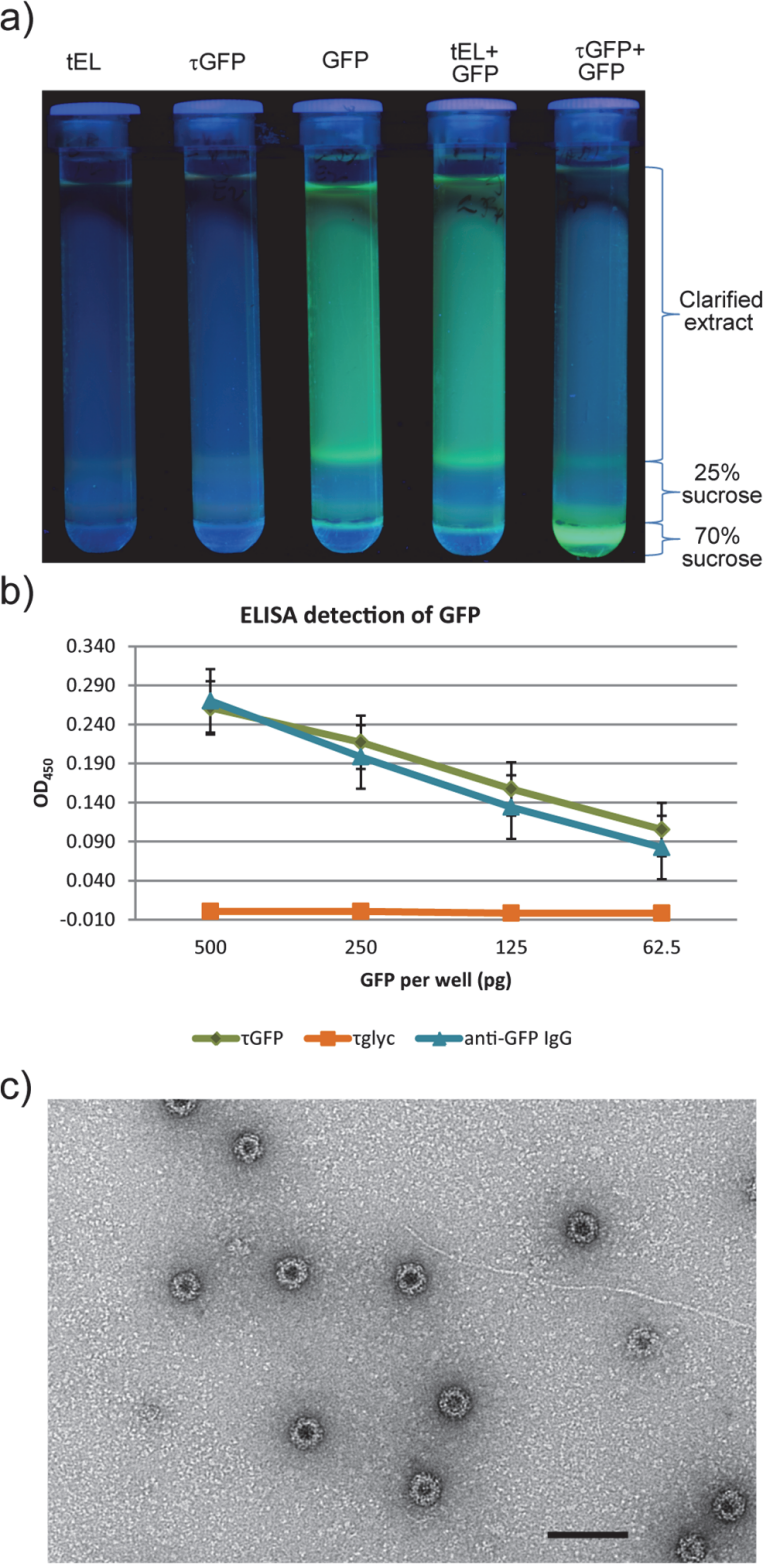
Plant-produced τGFP particles bind GFP. a) Ultracentrifuge tubes containing sucrose cushions photographed under UV light after ultracentrifugation. GFP-associated fluorescence remains in the supernatant when GFP-containing plant lysate is centrifuged alone or mixed with tEL-containing plant lysate; but migrates through the cushion when GFP-containing and τGFP-containing plant lysates are mixed. b) Detection of GFP by sandwich ELISA, after coating wells with τGFP (green), τglyc (orange) or an anti-GFP polyclonal IgG (blue) and adding GFP to the wells at four different concentrations after blocking. Detection is horseradish peroxidase—mediated ECL, and signal is net of background. Error bars are standard error. c) Electron micrograph of plant-produced τGFP particles in the presence of GFP, purified by sucrose cushion and size exclusion chromatography. Scale bar 100 nm.

The τGFP particles were shown to bind to GFP by two different methods. Firstly, plant tissue expressing either the τGFP particles or GFP were homogenised together before being loaded on a sucrose cushion. After ultracentrifugation, the fluorescence associated with GFP sedimented to the bottom of the sucrose cushion, co-localising with the τGFP particles (Fig. 8A). When plant extracts containing either GFP alone or mixed extracts containing GFP and tEL “empty-loop” tandem core particles were similarly centrifuged through sucrose cushions, the fluorescence remained in the supernatant. These results indicate that GFP binds specifically to, and co-sediments with, τGFP, but not tEL particles. GFP binding to τGFP was also demonstrated by a modified sandwich ELISA using wells coated with the τGFP particles. As a negative control, wells were coated with the same amount of a different but similarly-produced tandibody particle, which displays a nanobody against a viral glycoprotein (τglyc). The positive control was a commercially available anti-GFP polyclonal antibody different to that used for detection. A dilution series (Fig. 8B) indicated that the binding of GFP to wells coated with τGFP or with anti-GFP antibody was very similar; by contrast, the negative control showed no signal above background. These results indicated that the interaction between τGFP and GFP is caused specifically by the anti-GFP nanobody moiety in the tandibody. The appearance of the τ-GFP particles bound to GFP was analysed by negative stain electron microscopy (Fig. 8C).

**Fig 9:**
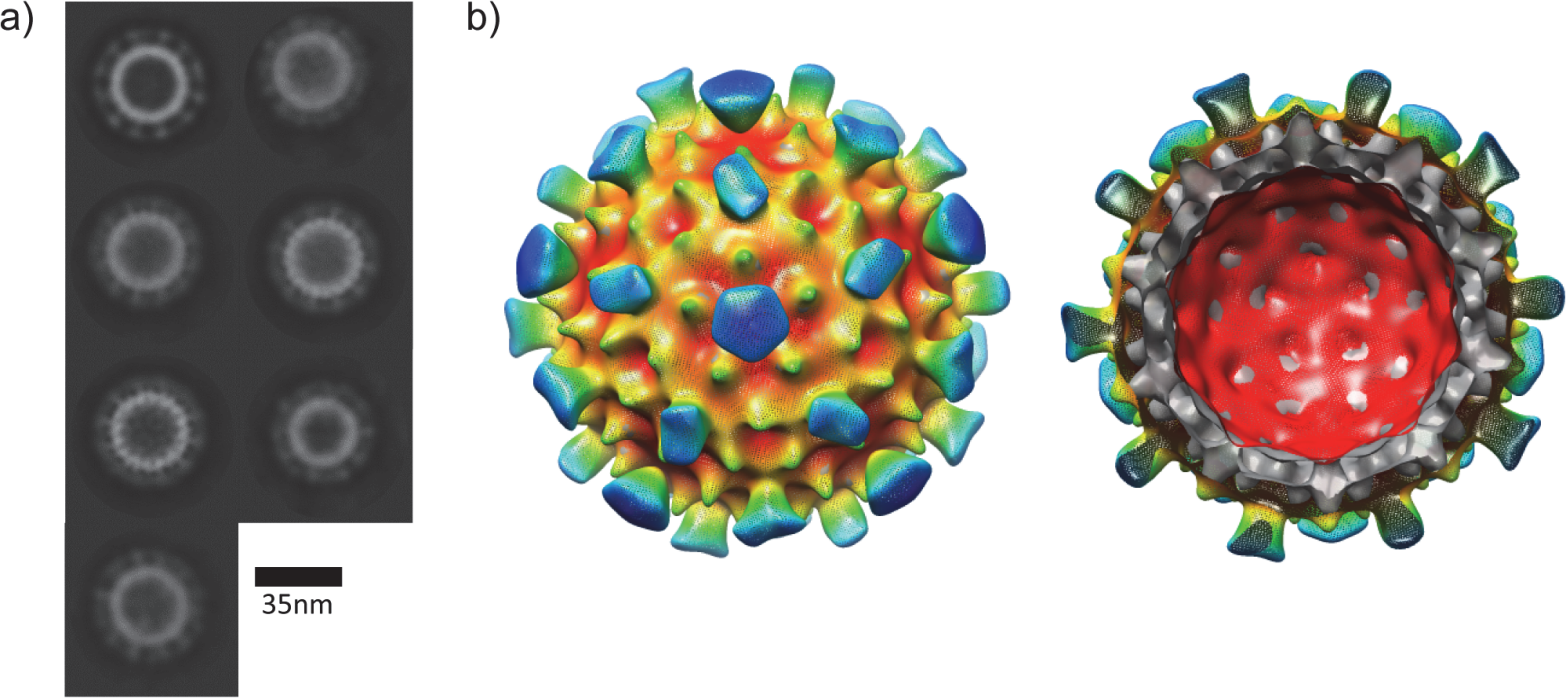
Cryo-EM of plant-produced τGFP bound with GFP. a) Class averages computed using Relion of the τGFP particles. b) A 3D reconstruction (resolution estimate 25Å using the “gold standard” cross-FSC at cutoff 0.143) coloured by distance from the centre of the particle (red to blue). The map is shown viewed down a 5-fold axis with the He reconstruction on which the construct was based fitted within (grey surface). The projecting spikes represent density arising from the bound nanobody and GFP but do not occupy every position expected, instead appearing as an average of the density present with the highest intensity at the 2-fold (pseudo- 6-fold) axes and also at the 5-fold axis. These spikes are to some extent artefacts of the icosahedral symmetry imposed on the maps, but are reflected in the spikes also shown in the unaveraged class averages shown in a).

Finally, τGFP cores with GFP bound were subjected to cryo-EM as the CoHe and CoHe-GFP samples had been previously. From this we obtained class averages in which projecting spikes of density could easily be discerned around the edge of the HBV core surface (Fig. 9A), which 3D reconstruction showed to derive from the incorporated nanobody and bound GFP (Fig. 9B). As with the CoHe-GFPL map, due to disorder the projecting densities do not follow the icosahedral symmetry of the reconstruction and are therefore not observed projecting discretely from each core spike. Although they do show a coalescence of density around the 2- and 5-fold axes that may indicate some preferential orientations on the capsid surface which is reproduced in the class averages, the map density at this radius from the centre becomes more-or-less continuous when contoured at a lower level. It is worth noting that the spikes projecting from the capsid surface are located at the 2-fold axes in both the CoHe-GFPL and τGFP + GFP maps; in contrast while satellite density is located at the 5-fold axes in the τGFP maps it circles them in the CoHe-GFPL map. In any case, it is clear that the nanobodies bound with GFP project from the surface of the capsid and that they have not interfered with the assembly of the underlying core.

## Discussion

The results described here show that HBcAg proteins fused via a linker sequence produce covalently linked dimeric core proteins capable of assembling into particulate structures resembling the native HBc particles. The tandem core particles have been successfully purified using methods that are routinely used for monomeric HBcAg capsids. Cryo-EM analysis of the hetero-tandem core particles that comprise a C-terminally truncated core covalently linked to a full length core sequence yields a structural model that is almost identical to truncated monomeric core particles. The difference between the two models (homo-tandem and hetero-tandem) can be accounted for by the presence of additional C-terminal sequence in the second core of the hetero-tandem core protein.

Numerous studies have demonstrated the strong inherent immunogenicity of the HBc particle [1,7,8,11]. This is at least in part due to the multimeric presentation of antigenic determinants on the surface of the particle and the presence of several strong helper T cell epitopes within the HBcAg protein sequence. In addition to these inherent immunogenic features, the folding and self-assembly properties of the HBcAg protein are extremely robust and can function following the insertion of a wide range of foreign sequences in at least three positions in the primary sequence of the protein. These are the N-terminus, several positions towards the C-terminus and an internal position, the MIR epitope, located at and around amino acid 80 [13,26]. Insertions at the N-terminus, the MIR and towards the C-terminus (providing the terminal protamine-like domain is deleted) all appear at the surface of assembled particles [15,27–30]. However, the MIR site is the most exposed and the majority of the antibodies induced by HBc particles recognise this region [12]. Furthermore, the immunodominance of this antigenic site is transferred to foreign sequences inserted at that position [12,26,31,32]. For these reasons recombinant HBcAg protein is an attractive carrier system for the development of vaccines designed to induce high levels of specific antibodies. It also has potential as a convenient system for the study of receptor/ligand interactions, as has been demonstrated for the interaction between integrin molecules and the receptor binding domain of foot-and-mouth disease virus [31].

The close proximity of the MIRs of adjacent HBcAg proteins in the dimers, which form the primary building blocks of the icosahedral particle, is a potential restriction to the full exploitation of the benefits of the system. Interactions between adjacent inserts may adversely influence their adopting appropriate conformations, and the size of permitted insertions is likely to be restricted. Although particles were formed from HBcAg protein in which the green fluorescent protein (GFP) had been inserted at the MIR, this required the incorporation of very long flexible linker sequences and yields of recombinant particles were low [33].

In *E*. *coli*, significantly higher yields of particles were obtained with the homo-tandem cores compared to hetero-tandem cores, but in both *E*. *coli* and plants the hetero-tandem particles were more homogeneous than their homo-tandem counterparts. In addition, the hetero-tandem constructs were more tolerant of inserted foreign sequence than the homo-tandem equivalents. HBcAg protein, which is responsible for HBV genome packaging *in vivo* [34], can also exhibit RNA-binding features under conditions of heterologous expression [35]. Therefore, these particles have potential as both gene delivery vectors and in antigen presentation [36].

Electron microscopic examination showed that tandem HBcAg particles produced in plants are more structurally uniform than those produced in *E*. *coli*, perhaps due to a more favourable environment for accurate protein folding in the eukaryotic system. *N*. *benthamiana* in concert with the pEAQ vector system is a suitable method for the production of large quantities of tandem core particles. These particles could be produced with “empty” MIR loops, or with whole-protein inserts in the core 2 MIR. This was demonstrated with a direct fusion of GFP to the surface of the tandem particles, as well as with the fusion of a camelid-derived nanobody to produce tandibodies, which not only allowed the formation of VLPs, but maintained the binding activity of the nanobody moiety. This opens the door for non-covalent binding of any protein of interest to the surface of a tandibody scaffold, so long as a suitable nanobody with the appropriate specificity can be found. Incorporation of a nanobody specific for a defined peptide sequence may facilitate the use of such a tandibody as a universal presentation system for suitably tagged proteins. Investigations into this area are underway.

It is worth noting that non-covalent attachment of antigens to the HBcAg VLP is not an entirely new idea: an oligopeptide with affinity for the MIR of HBcAg has previously been used to display the extracellular domain of influenza M2 protein on the surface of HBcAg [37]. However, the binding between the oligopeptide and VLP broke down during ultracentrifugation on a sucrose cushion. It is also important to note that we attempted antibody-display on the surface of tandem cores in plants with the more commonly used single-chain variable fragment antibody (scFv) as opposed to single-domain VHH, but expression yields were extremely low and no particle formation was observed. This is most likely due to the more complicated structure of scFv, which have two domains corresponding to the variable regions of the light and heavy chains, whereas nanobodies only have a single domain corresponding to the variable region of the heavy chain [38,39]. Our results also revealed that nanobodies could not be displayed on the surface of monomeric (i.e. non-tandem) HBc particles in plants.

The data shown here demonstrates that an insert of 27 kDa, GFP, can readily be displayed on the surface of tandem cores. However, scFv, which are of a similar size, did not allow particle formation when fused into core 2. This indicates that size is not the only factor involved in determining whether an insert can be displayed on the surface of a tandem core VLP: amino acid composition, and the distance between the N- and C- termini of the insert, also probably play a major role. It is therefore likely that some proteins larger than 27 kDa will be successfully displayed on the surface of tandem cores, while certain other proteins of much smaller size will not allow particle formation. The constructs presented here all contain the heterologous insert in core 2. The constructs were designed in this way on the assumption that an unmodified core 1 would fold easily and promote proper folding of the modified core 2 during the translation process. Future work will include modifying core 1 and both cores simultaneously.

To conclude, we have shown that two copies of the HBcAg protein, when fused in tandem, produce covalently linked dimers able to assemble into VLPs very similar in structure to wild-type HBc VLPs when expressed in both bacterial and plant expression systems. Moreover, we have shown that such tandem core particles allow the display of correctly-folded heterologous proteins on the surface of assembled HBc VLPs. Furthermore, tandem core constructs incorporating foreign proteins can produce assembly-competent protein in plants, even when such fusions are not functional in a monomeric core system. We have further shown that a nanobody displayed on tandem core particles retains its ability to bind the cognate antigen, thereby opening the door to the non-covalent binding of proteins of interest to the tandibody scaffold. Further work will explore the use of tandem cores, and particularly tandibodies, as tools for antigen display for vaccine design.

## Materials and Methods

### Construction of tandem HBcAg protein expression vectors

Tandem HBcAg sequences were constructed by overlapping PCR with different numbers of glycine-rich (GGS) repeats linking two copies of the HBcAg sequence. The upstream HBcAg protein (core 1) was amplified as a C-terminally truncated molecule terminating at amino acid 149. The downstream copy (core 2) was amplified as either the full-length protein or as a similar truncation (Fig. 1B). These were termed hetero-tandem (He), and homo-tandem (Ho) respectively. Enzymes and buffers used throughout all cloning steps were supplied by New England Biolabs.

### Expression of HBV tandem core proteins in *E*. *coli*

The sequenced expression clones were transferred to *Escherichia coli* BL21 DE3 cells for protein production. Following successful demonstration of particle production from tandem core constructs, the sequences were codon-optimised to eliminate codons not frequently found in the bacterial genome. The sequence was analysed using the online Graphical Codon Usage Analyser [40] and silent mutations were introduced where codons infrequently used by *E*.*coli* were found, restriction sites were placed in the MIR loop and flanking each core coding sequence to facilitate manipulation of the construct and insertion of foreign antigens. Seven repeats of the GGS motif (GGS)_7_ were placed between the two core sequences. The final, codon-optimised (Co) constructs were termed CoHo and CoHe.

### Insertion of GFP into MIR site of core 2

Two insertion sequences based on enhanced GFP (eGFP), with either GGS or GGGGSGGGG linkers were prepared by PCR amplification. Following digestion with EcoRI and NheI these were inserted into the MIR of the second core in the hetero-tandem core sequence (CoHe) to give CoHe-GFPs (with short linkers) and CoHe-GFPL (with long linkers).

### Purification of tandem core particles from bacterial lysates

Transformed and induced bacterial cells (BL21) were pelleted by centrifugation at 3500 x g in a Sorvall SLA 1500 rotor for 20 min at 4°C. Supernatant was discarded and bacteria resuspended in 1 x lysis buffer (20 mM HEPES pH 7.5, 250 mM NaCl, 5 mM DTT, 1 x protease inhibitor cocktail [Roche], +/- 10 mM EDTA). Cells were lysed by two passes through a French Press at 14,000 psi. A non-specific nuclease (Benzonase, Sigma) was added to the lysate at 16 units per ml after each pass through the French Press, and lysates were then sonicated. The resulting suspensions were clarified by centrifugation at 26,000 x g for 20 min at 4°C in a Sorvall SS-34 rotor and the supernatants were designated as the soluble fraction and used for further purification.

The soluble fractions were layered over 10 ml cushions of 30% sucrose in buffer a (20 mM HEPES pH 7.5, 250 mM NaCl) and centrifuged at 150,000 x g for 3 h at 8°C in a Sorvall AH629 rotor and pellets were resuspended in 6 ml of buffer a. Pellet suspensions were sonicated before clarification at 26,000 x g for 20 min at 4°C in a Sorvall SS34 rotor and the supernatant was loaded directly onto a discontinuous sucrose gradient (5 ml increments of 60, 50, 40, 30, 20, and 10% sucrose in buffer a). Sucrose gradients were centrifuged at 150,000 x g for 3 h at 18°C.

Fractions were collected from the bottom of the tube and analysed by Bradford assay (total protein content), SDS page (size and purity), and by electron microscopy for the presence of VLPs.

### Construction of plant expression vectors

PCR was used to amplify the sequences corresponding to constructs CoHe, CoHo, CoHe-GFPs, and CoHe-GFPL, along with the Δ176 monomeric HBcAg gene, a C-terminal truncation at amino acid 176 of the HBcAg gene described in Meshcheriakova et al. [41]. The resulting amplified DNA was transferred to the plant transient expression vector pEAQ-*HT* [23,42] either by restriction enzyme or Gateway-based cloning to give plasmids pEAQ-CoHe, pEAQ-CoHo, pEAQ-CoHe-GFPs, pEAQ-CoHe-GFPL, and pEAQ-Δ176. To generate a plant-based tandem core expression system suitable for rapid manipulation in the pEAQ-*HT* plasmid, a new version of the CoHe construct, named tEL, was synthesised by GeneArt (Life Technologies) and inserted into the pEAQ-*HT* vector to give pEAQ-tEL. This tandem construct contains a polylinker with unique restriction sites separated by linker sequences in each MIR loop: PGAGGSSGQL in core 1, and VDAGGGPRGGSGIN in core 2; where the underlined pairs of amino acids correspond to restriction sites XmaI, MfeI, SalI, AvrII, an AseI, respectively.

The amino acid sequence of an alpaca-derived anti-GFP single-domain antibody (VHH, or nanobody) was obtained from GenBank protein database (accession 3K1K_C, as described by Kirchhofer et al. [25]) and the gene was synthesised by GeneArt (Life Technologies) with codon usage optimised for expression in *N*. *benthamiana*. The anti-GFP VHH gene was then cloned in-frame into the MIR loop of core 2 of pEAQ-tEL using the SalI and AseI restriction sites, as well as the MIR loop of a monomeric HBcAg construct, named pEAQ-mEL, which is identical to the C-terminal core of tEL. In both cases, a (GGGGS)_3_ linker was added on each end of the anti-GFP VHH sequence, and the resulting tandem and monomeric constructs were named pEAQ-τGFP, and pEAQ-μGFP, respectively.

All expression constructs were transformed into *Agrobacterium tumefaciens* LBA4404 by electroporation and propagated at 28°C in Luria–Bertani media containing 50 μg/ml kanamycin and 50 μg/ml rifampicin. Transient expression was carried out by agroinfiltration of 3–4 week old *N*. *benthamiana* leaves as described by Thuenemann et al. [43].

### Extraction of HBcAg proteins from plant tissue

To examine the expression levels of the various tandem core constructs, small scale extracts were prepared from leaf material at various days post-infiltration (dpi). Leaf discs were disrupted using either a FastPrep (MP Biochemicals) or omni Bead Ruptor 24 (Camlab) homogenizer. The samples were analysed by SDS PAGE and western blotting either directly or after clarification by centrifugation at 16,000 g for 10 min.

To purify assembled particles derived from constructs pEAQ-Δ176, pEAQ-CoHe, pEAQ-CoHo, pEAQ-CoHe-GFPs, and pEAQ-CoHe-GFPL, leaf tissue was harvested 6 or 7 dpi and homogenised in three volumes of HBexB1 buffer (10 mM Tris-HCl pH 8.4, 120 mM NaCl, 1 mM EDTA, 0.75% (w/v) sodium deoxycholate, 1 mM DTT plus complete protease inhibitor cocktail (Roche). The extracts were clarified by centrifugation at 16,000 x g for 2 min at 4°C, loaded onto 30–60% (w/v) continuous sucrose gradients in buffer b (10 mM Tris-HCl pH 8.4, 120 mM NaCl) and centrifuged in a SW41Ti rotor (Beckman) at 273,800 x g for 2.5 h at 4°C. The gradient fractions were analysed by SDS-PAGE and western blotting. The purified particles were designated Δ176, CoHe, CoHo, CoHe-GFPs, and CoHe-GFPL.

To purify assembled particles derived from constructs pEAQ-tEL and pEAQ-τGFP, leaf tissue was harvested 7 dpi and homogenised in three volumes of buffer c (0.1 M sodium phosphate, pH 7, plus complete EDTA-free protease inhibitor cocktail tablets [Roche]), using a Waring blender. The crude extracts were filtered over miracloth (Calbiochem) and the filtrates clarified by centrifugation at 9,000 x g for 10 min at 4°C. The clarified extracts were then filtered through a minisart 0.45 μm syringe filter (Sartorius) and loaded over sucrose cushions consisting of consecutive layers of 2 ml of 25% (w/v) and 0.5 ml 70% (w/v) sucrose in buffer c in UltraClear 14X89 mm ultracentrifuge tubes (Beckman Coulter). Ultracentrifugation was carried out as above. The purified particles were designated tEL and τGFP.

### Demonstration of binding of soluble GFP to τGFP particles by co-sedimentation

To investigate the ability of the anti-GFP nanobody expressed on the surface of τGFP to bind to GFP, *N*. *benthamiana* leaves were infiltrated separately with pEAQ-tEL (with no heterologous protein in the MIR loops), pEAQ-τGFP (displaying the anti-GFP nanobody), pEAQ-GFP (expressing soluble GFP) or the empty pEAQ-*HT* vector. Leaves were harvested 7 dpi and GFP-expressing leaves mixed with an equal weight of those expressing the various HBc constructs. The leaves were then homogenised and the clarified extracts sedimented through a sucrose cushion as described above. The tubes were photographed under ultraviolet light immediately after ultracentrifugation.

Transmission electron microscopy of GFP-τGFP complexes was carried out on material produced by co-infiltrating *N*. *benthamiana* leaves with pEAQ-τGFP and pEAQ-GFP. The complexes were isolated as described above and further purified by chromatography over Sepharose CL-4B resin (Amersham Biosciences) in a XK 16 4/15 column. Fractions containing assembled particles were dialysed against 20 mM ammonium bicarbonate, pH 7.5, and concentrated on a Speed Vac (Savant) prior to electron microscope analysis.

### Analysis of τGFP binding to GFP by ELISA

Particles of τGFP and τglyc (a negative control construct identical to τGFP except for the nanobody moiety, which is replaced with a llama-derived nanobody specific to a viral glycoprotein) were produced and purified from plants as described above. These particles, along with a goat polyclonal antibody specific for GFP (Abcam ab6673; positive control), were used to coat the wells of a 96-well plate (F96 Maxisorp, Nunc) at 25 ng/well. After blocking, four serial two-fold dilutions of recombinant GFP (Millipore 14–392) were added to quadruplicate wells, and the GFP was detected using a horseradish peroxidase—conjugated anti-GFP antibody (Invitrogen A10260). The OD_450_ of the wells was read on a POLARstar Omega plate reader (BMG Labtech), and the average signal from control wells (with PBS added instead of GFP) was subtracted from the average signal from experimental wells.

### SDS-PAGE and Western blot analysis

Protein extracts were analysed by electrophoresis on 12.5% (w/v) polyacrylamide-SDS or NuPAGE Bis-Tris gels (Life Technologies). Western Blot analyses were performed using a monoclonal primary antibody against HBcAg protein (mAb 13 or 10E11 [Abcam]) followed by detection with a goat anti-mouse secondary antibody conjugated to horseradish peroxidase, and developed using the chemiluminescent substrate ECL^plus^ (Amersham Pharmacia).

### Negative-stain transmission electron microscopy

Particle preparations were spotted onto carbon-coated copper or copper-palladium grids and negatively stained with 2% (w/v) uranyl acetate. The grids were examined using a Philips CM10, Philips CM100 or Tecnai 20 transmission electron microscope.

### Cryo-transmission electron microscopy

Sucrose gradient fractions were buffer-exchanged for 10 mM Tris-HCl pH 8.0, 50 mM NaCl. Vitrified specimens were prepared on holey carbon grids using standard protocols. The bacterially-expressed particles were imaged on a CM120 BioTwin transmission electron microscope (FEI-Philips) using low-dose procedures at 100 kV and 37,000 x magnification on SO-163 film later digitised using a Zeiss-SCAI scanner with a step size of 7 μm. Scanned images were binned by a factor of 2, then visualized and individual particles interactively picked, using the MRC program suite [44]. Icosahedral reconstructions were determined using self-common lines procedures [45] to obtain initial models and then polar Fourier transform (PFT) protocols [46,47] in refinement. Resolution was estimated using Fourier shell correlation with FSC = 0.5 as a cut-off value [21] to indicate values of ~30 Å for both Ho and He. The reconstructions were Fourier transformed, merged and filtered to 30 Å and a difference map obtained using FSMELT and SHELLSCALE [48] and final maps calculated by back-transformation using FFT from the CCP4 program suite [49]. Figures were prepared using BOBSCRIPT [50–52].

Images of the CoHe-GFPL particles were obtained using a Tecnai F30 microscope at 300 kV and 39,000 x magnification and for a range of defocus values from −4 to −8 μm. Images were captured on Kodak S0–163 film (scanned as above with a 7 μm step size). Following CTF correction in EMAN [53] reconstruction of the CoHe-GFPL images was achieved using IMAGIC and SPIDER software; 441 images were reconstructed using icosahedral symmetry to a resolution of ~36 Å. Images of the τGFP + GFP particles were obtained using the same F30 microscope but operating at 200 kV and collecting the data on a Falcon 2k CCD camera with a magnification of 80,000 x giving a sampling on the object scale of 2 Å and for defocus values from −2 to −5 μm. After manual selection in EMAN [53], particles were corrected for contrast transfer function, classified in two dimensions and reconstructed in 3D using RELION software [54], the initial 3D model being generated with IMAGIC software from seven RELION-derived class averages [55]. By these means 2,385 images of τGFP + GFP were reconstructed at 25 Å resolution (“gold-standard” cross-FSC cutoff 0.143), reaching convergence after thirteen iterations.
